# Discriminative Value of Serum Irisin in Prediction of Heart Failure with Different Phenotypes among Patients with Type 2 Diabetes Mellitus

**DOI:** 10.3390/cells11182794

**Published:** 2022-09-07

**Authors:** Alexander A. Berezin, Michael Lichtenauer, Elke Boxhammer, Eric Stöhr, Alexander E. Berezin

**Affiliations:** 1 Internal Medicine Department, Zaporozhye Medical Academy of Postgraduate Education, 20, Vinter Av., 69096 Zaporozhye, Ukraine; 2 Department of Internal Medicine II, Division of Cardiology, Paracelsus Medical University Salzburg, 5020 Salzburg, Austria; 3 COR-HELIX (CardiOvascular Regulation and Human Exercise Laboratory—Integration and Xploration), Leibniz University Hannover, 30167 Hannover, Germany; 4 Internal Medicine Department, Zaporozhye State Medical University, 26, Mayakovsky Av., 69035 Zaporozhye, Ukraine

**Keywords:** heart failure with preserved ejection fraction, heart failure with mildly reduced ejection fraction, heart failure with reduced ejection fraction, type 2 diabetes mellitus, irisin, natriuretic peptides, prediction

## Abstract

Recent studies have shown that circulating levels of irisin are prognostic factors in heart failure (HF), but no data are available on the predictive role of irisin in patients with type 2 diabetes mellitus (T2DM) and different phenotypes of HF. The aim of the study was to investigate whether serum levels of irisin predict HF in T2DM patients. We prospectively included 183 participants with T2DM aged 41 to 62 years (30 non-HF patients and 153 HF patients) and 25 healthy volunteers in the study and evaluated clinical data, hemodynamics and biomarkers (N-terminal pro-brain natriuretic peptide (NT-proBNP) and irisin). Serum levels of irisin < 8.30 ng/mL were found to be a better indicator of HF with reduced ejection fraction (HFrEF) than irisin ≥ 8.30 ng/mL, but the predictive cut-off point for NT-proBNP remained the same as for HF with mildly reduced ejection fraction (HFmrEF). Serum levels of irisin < 10.4 ng/mL significantly improved the predictive ability of NT-proBNP for HF with preserved ejection fraction (HFpEF). In conclusion, we found that decreased serum levels of irisin significantly predicted HFpEF, rather than HFmrEF and HFrEF, in T2DM patients. This finding may open a new approach to HF risk stratification in T2DM patients.

## 1. Introduction

Heart failure (HF) remains an important factor influencing cardiovascular (CV) mortality and the risk of hospitalization or readmission in patients with known CV diseases [1]. Although the worldwide prevalence of HF with reduced (HFrEF) and mildly reduced (HFmrEF) ejection fraction remained stable over the past decade, HF with preserved ejection fraction (HFpEF) has shown a steady increase, directly related to the aging population and the increase in relevant comorbidities such as type 2 diabetes mellitus (T2DM), chronic kidney disease and hypertension [2,3]. However, previous clinical studies have clearly provided evidence that the mortality risk among patients with different HF phenotypes shows a distinct resemblance [4,5]. In addition to clinical status and imaging findings that reveal adverse cardiac remodeling and altered cardiac systolic and diastolic functions, circulating biomarkers—particularly N-terminal natriuretic peptide type B (NT-proBNP)—seem to be reliable tools for risk stratification, diagnosis and treatment of HF [6]. At the same time, the discriminatory power of natriuretic peptides (NPs) is considered to be better in HFrEF than in HFpEF [7]. Indeed, lowering NT-proBNP during medical treatment was strongly associated with reversal of cardiac remodeling and improvement of clinical outcomes and CV mortality in HFrEF compared with HFpEF [8,9]. In this context, identifying new biomarkers with the aim of improving the discriminatory value of the current predictive model without an extensive increase in its total costs is of great value [10].

Irisin is a multi-functional peptide generated by proteolytic cleavage of the fibronectin type III domain-containing 5-transmembrane protein, whose expression is under tight control of the peroxisome proliferator-activated receptor-γ (PPAR-γ) coactivator 1α [11]. Irisin appears to be a myokine produced by both skeletal muscle and adipose tissue cells [12]. Under physiological and pathological conditions, irisin binds to widely distributed transmembrane αV/β5-integrins that act as its receptors and exerts numerous tissue trophic biological effects. These include the reduction of systemic inflammation, the modulation of bone resorption/formation balance, lipid, glucose and energetic metabolism, thermogenesis, browning of white adipose tissue, as well as functioning of the nervous system through the activation of p38 MAP kinase, AMPK/PI3K/Akt/ERK1/2 and STAT3/Snail signaling pathways and downregulation of the mTOR pathway [13,14,15,16].

Irisin suppresses the expression of proinflammatory genes and decreases the release of proinflammatory cytokines in patients with abdominal obesity and T2DM, thereby attenuating adipose tissue and vascular inflammation and improving insulin resistance, liver function and endothelial function [17,18]. In addition, irisin suppresses cardiomyocyte apoptosis, reduces cardiac hypertrophy and oxidative stress, protects against ischemia and reperfusion injury and promotes vasodilation, as well as paracrine angiogenic and antifibrotic effects on the myocardium and the vessels [19,20]. There is evidence that irisin levels are sufficiently lower in patients with T2DM than in non-T2DM patients, regardless of the presence of coronary artery disease (CAD), severity of coronary artery atherosclerotic lesions and development of collaterals [21,22]. Recent studies have shown that chronic HFrEF patients have lower irisin levels than non-HF patients regardless of the etiology of the disease [23,24], whereas acute HF patients with higher serum irisin levels have a significantly worse survival rate than those with lower levels [25]. Little is known about the predictive role of irisin in T2DM patients with different phenotypes of HF [26]. Therefore, the aim of this study was to investigate whether serum levels of irisin predict different phenotypes of HF in patients with T2DM.

## 2. Materials and Methods

### 2.1. Study Design and Cohorts of Participants

**Figure 1:**
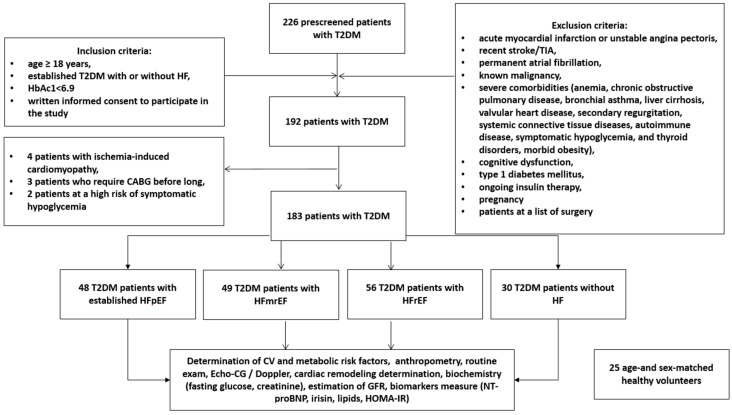
Flow chart of the study design. Abbreviations: CV, cardiovascular; GFR, glomerular filtration rate; HFpEF, heart failure with preserved ejection fraction; HFmrEF, heart failure with mildly reduced ejection fraction; HFrEF, heart failure with reduced ejection fraction; HOMA-IR, Homeostatic Assessment Model of Insulin Resistance; HbA1c, glycosylated hemoglobin; NT-proBNP, N-terminal brain natriuretic pro-peptide; T2DM, type 2 diabetes mellitus; TIA, transient ischemic attack.

A total of 183 participants with T2DM aged 41 to 62 years (30 non-HF patients and 153 HF patients) were prospectively recruited for the study from October 2020 to December 2021. The T2DM patients were treated in the private hospital Vita-Centre (Zaporozhye, Ukraine). The healthy control group consisted of 25 individuals matched with age and sex to all other groups of patients. The following inclusion criteria were used: age ≥ 18 years, established T2DM with or without HF, adequate control for hyperglycemia (HbAc1 < 6.9%), written consent to participate in the study. Exclusion criteria were acute myocardial infarction or unstable angina pectoris, recent stroke/transient ischemic attack (TIA), permanent atrial fibrillation, known malignancy, severe comorbidities (anemia, chronic obstructive lung disease, bronchial asthma, liver cirrhosis, known valvular heart disease, symptomatic hypoglycemia, morbid obesity, congenital heart disease, systemic connective tissue diseases, autoimmune disease, cognitive dysfunction and thyroid disorders), type 1 diabetes mellitus, ongoing insulin therapy and pregnancy. All healthy participants had no history of CV disease. Figure 1 illustrates the flow chart of the study design.

### 2.2. Determination of Patients’ Background, Risk Factors and Comorbidities

HF, including HFrEF (LVEF < 40%), HFmrEF (LVEF = 40–49%) and HFpEF (LVEF ≥ 50%), was detected according to ESC criteria [27,28]. The criteria for HFpEF were: (1) presents with symptoms and/or signs compatible with HF; (2) EF > 50%; (3) functional and structural alterations are an average mitral E/e’ ratio ≥ 13 and an average septal-lateral e’ velocity < 9 cm/s; LV mass > 115/95 g/m^2^ men/women or LAVI > 34 mL/m^2^ [27,28]. To determine T2DM [29], dyslipidemia [30] and hypertension [31], the guidelines that were valid at the current time of enrollment were used. Severe anemia has been referred as reduced levels of Hb < 80.0 g/L for both genders. Morbid obesity was determined as a body mass index (BMI) of 40 kg/m^2^ or higher.

### 2.3. Anthropometric Measurements and Clinical Examinations

All patients enrolled in the study underwent general clinical and physical examination. We also measured office blood pressure (BP), heart rate, height, weight, waist circumference, hip-to-waist ratio (WHR) and BMI.

### 2.4. Concomitant Medications

T2DM was treated with a personally adjusted dose of metformin and diet. T2DM patients with HF additionally took sodium-glucose cotransporter-2 (SGLT2) inhibitor (empagliflozin 10 mg daily or dapagliflozin 10 mg daily), whereas non-HF patients were optionally treated with SGLT2i. Hypertension therapy was executed with ACE inhibitor (ACEI) or angiotensin-II receptor blocker (ARB). Thiazides were added when needed to reach a blood pressure control (office BP < 140/90 mmHg and/or average daily BP < 130/80 mm Hg). Lipid-lowering medication—mainly rosuvastatin (20–40 mg daily)—were used in all patients with dyslipidemia, T2DM at high CV risk or known CVD without conventional contraindications. Beta-blockers in individually adjusted optimal daily dose along with mineralocorticoid receptor antagonist, ACEI or angiotensin receptor neprilysin inhibitor (ARNI) were prescribed for HFrEF/HFmrEF patients. HFpEF patients were treated with a combination of beta-blockers and ACEI/ARB. Loop diuretics (furosemide, torasemide) were used when fluid retention was determined. Acetylsalicylic acid (75 mg daily) or clopidogrel (75 mg daily) were also prescribed as concomitant medications.

### 2.5. Echocardiography and Doppler Method

B-mode transthoracic echocardiography was carried out at study entry with the diagnostic system Vivid T8 (GE Medical Systems, Freiburg, Germany) using a 2.5–3.0 MHz phase probe. Hemodynamic parameters were determined in accordance with current recommendation of the European Association of Cardiovascular Imaging (EACVI) and the American Society of Echocardiography (ASE) [32]. Left ventricular (LV) ejection fraction (LVEF) was measured using the Simpson method [33]. Left atrial volume was directly measured and then the left atrial volume index (LAVI) and E/e’ ratio were estimated [33]. The E/e’ ratio was estimated as a ratio between early mitral inflow velocity and mitral annular early diastolic velocity given as averaged septal and lateral e’. LV hypertrophy (LVH) was determined by conventional Echo criteria (LV mass/body surface area ≥ 125 g/m^2^ in male or ≥ 110 g/m^2^ in female) [33]. In addition, the left ventricle myocardial mass index (LVMMI) was calculated according to the current recommendation [33].

### 2.6. Estimating Glomerular Filtration Rate

Glomerular filtration rate (GFR) was calculated using the CKD-EPI formula [34].

### 2.7. Insulin Resistance Determination

Insulin resistance was evaluated by the Homeostatic Assessment Model of Insulin Resistance (HOMA-IR) using the conventional equation [35]: HOMA-IR = fasting insulin (mU/L) × fasting glucose (mmol/L)/22.5.

### 2.8. Blood Sampling and Biomarker Measurements

Blood samples were drawn in the morning following overnight fasting (at 7–8 a.m.) into barcoded silicone test tubes. Then, samples were centrifuged upon permanent cooling at 6000 rpm for 3 min. Afterwards, the plasma was immediately refrigerated. Each aliquot was stored at a temperature of −70 °C. In order to measure the levels of glycosylated hemoglobin (HbA1c), fasting glucose and insulin, total cholesterol (TC), low-density lipoprotein (LDL-C) cholesterol, high-density lipoprotein (HDL-C) cholesterol and triglycerides (TG), a Roche P800 analyzer (Basel, Switzerland) was used. Commercial ELISA kits produced by Elabscience (Houston, TX, USA) were used to determine levels of irisin and NT-proBNP according to the manufacturer’s recommendations. A Labline-90 analyzer (Frankenmarkt, Austria) and an Elecsys 1010 analyzer (F. Hoffmann-La Roche Diagnostics, Mannheim, Germany) were used, respectively, for the measurements.

### 2.9. Statistics

V. 23 Statistical Packages for Social Sciences (SPSS; IBM, Armonk, New York, NY, USA) software and v. 9 GraphPad Prism (GraphPad Software, San Diego, CA, USA) software for statistical analysis were used. Power Analysis and Sample Size (NCSS Statistical software, Kaysville, UT, USA) software was determined to calculate the sample size. The test level α was defined as 0.05 and the degree of certainty 1-β was 0.90, so that the preliminary sample size became 180.

Continuous variables with normal distribution were characterized by mean ± standard deviation (SD), whereas continuous, non-normally distributed variables were specified by median (Me) and interquartile range (IQR). The chi-square test was applied for non-continuous variables. Normal distribution of variables was checked with the Kolmogorov–Smirnov test. One-way analysis of variance (ANOVA) and Tukey’s post hoc test were used for the comparison between different groups. An F test was provided for the comparison within groups. Spearman’s correlation coefficient was calculated to ascertain the relationship between variables and then represented with the heat map plot. ROC curves with a separate analysis of the Youden Index were constructed to assess the reliability of the predictive models. Predictors for HF were determined by univariate and multivariate logistic regression analysis. An odds ratio (OR) and 95% confidence interval (CI) were reported for each predictor. Predictors of HF were confirmed using integrated discrimination indices (IDI) and net reclassification improvement (NRI). Differences were considered significant at the level of statistical significance *p* < 0.05.

## 3. Results

### 3.1. General Characteristics of the Patients Included in the Study

Table 1 illustrates basic characteristics of the patients included in the study. The entire patient cohort was composed of mainly males (64.5%) with an average age of 51 who had several comorbidities and conventional CV risk factors, such as abdominal obesity (45.9%), hypertension (86.3%), dyslipidemia (83.1%), smoking (48.6%) and LV hypertrophy (78.7%). The control patients were matched on age and gender, but not on confounding CV risk factors. Therefore, patients from the entire population had higher WHR, LVEDV, LVESV, LVMMI, LAVI and E/e’ and lower LVEF than healthy volunteers. However, we did not find any differences in SBP and DBP between T2DM from the entire cohort and healthy volunteers.

We also noticed that there were no significant differences between T2DM patients in age, gender and prevalence of CV risk factors, including dyslipidemia, hypertension, smoking, abdominal obesity, as well as some anthropomorphic parameters (WHR), apart from LV hypertrophy, which occurred more often in HFpEF patients. Therefore, the proportion of the patients with New York Heart Failure (NYHA) classes II/III were similar in HFrEF, HFmrEF and HFrEF. Additionally, the patients with HFrEF had higher LVEDV, LVESV, LAVI and E/e’, and lower LVEF, compared with HFpEF and non-T2DM patients, whereas there was a similarity in these parameters in patients with HFpEF and HFmrEF, apart from in LVEF.

T2DM patients from the entire population demonstrated lower eGFR, HOMA-IR and HDL cholesterol, and higher levels of NT-proBNP, fasting glucose, HbA1c, creatinine, triglycerides and LDL cholesterol than healthy volunteers. No significant differences among T2DM cohorts in HOMA-IR, serum levels of creatinine, fasting glucose and lipids were detected.

Along with this, all HF patients were treated with SGLT2 inhibitors and the majority of them received any blocker of the renin–angiotensin–aldosterone system as concomitant medication, whereas non-HF T2DM patients were optionally treated with SGLT2 inhibitor and ACEIs/ARBs/ARNI were prescribed in 26 individuals (86.7%).

### 3.2. Circulating Levels of Irisin in T2DM Patients and Healthy Volunteers

**Figure 2:**
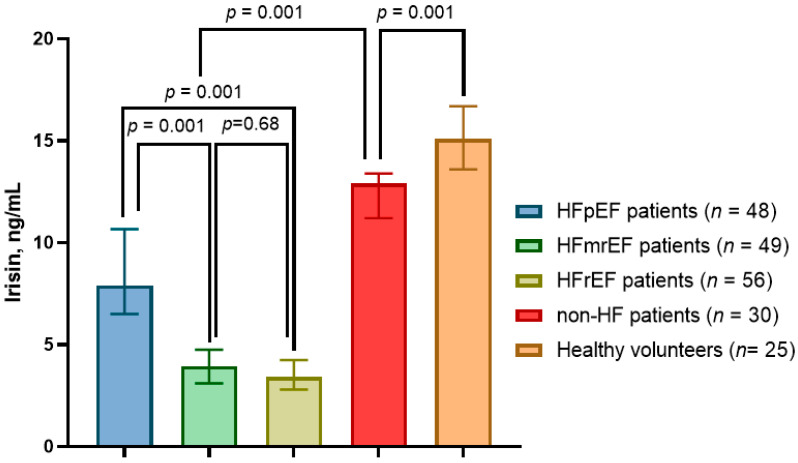
Levels of irisin in T2DM patients depending on HF phenotypes in comparison with healthy volunteers.

The levels of irisin were significantly higher in HFpEF patients than in HFrEF individuals (7.90 ng/mL; 95% CI = 6.85–10.66 ng/mL vs. 3.41 ng/mL; 95% CI = 2.80–4.24 ng/mL, *p* = 0.001) and were also higher than in HFmrEF patients (3.95 ng/mL; 95% CI = 3.10–4.75 ng/mL, *p* = 0.001), whereas non-HF T2DM patients (12.9 ng/mL; 95% CI = 11.2–13.4 ng/mL) demonstrated lower levels of irisin when compared to healthy volunteers (15.1 ng/mL; 95% CI = 13.6–16.7 ng/mL; *p* = 0.001) (Figure 2).

### 3.3. Spearman’s Correlation between Irisin Level and HOMA Index, NT-proBNP, Lipid Profile and Hemodynamics Parameters

**Figure 3:**
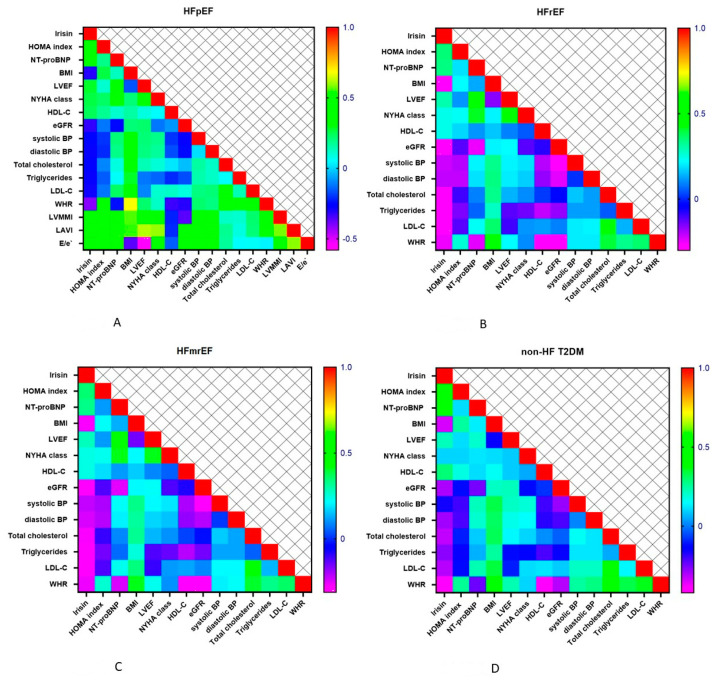
Heat map plots comparing serum levels of irisin with other variables in different cohorts of patients with T2DM. (**A**): Heat map for HFpEF; (**B**): Heat map for HFrEF; (**C**): Heat map for HFmrEF; (**D**): Heat map for non-HF T2DM. Abbreviations: BMI, body mass index; BP, blood pressure; eGFR, estimated glomerular filtration rate; LDL-C, low-density lipoprotein cholesterol; HDL-C, high-density lipoprotein cholesterol; HF, heart failure; HOMA-IR, Homeostatic Assessment Model of Insulin Resistance; HFpEF, heart failure with preserved ejection fraction; HFmrEF, heart failure with mildly reduced ejection fraction; HFrEF, heart failure with reduced ejection fraction; LVEF, left ventricular ejection fraction; NT-proBNP, N-terminal brain natriuretic pro-peptide; NYHA, New York Heart Association; T2DM, type 2 diabetes mellitus; WHR, waist-to-hip ratio.

The correlations of irisin levels and the HOMA index, NT-proBNP, lipid profile and hemodynamics parameters in patients with all phenotypes of HF are reported in Figure 3A–D. The heat maps graphically represent the most valuable positive correlation of the levels of irisin with NT-proBNP, LVEF, NYHA class and the HOMA index, and the inverse correlation with BMI and WHR regardless of HF phenotypes.

### 3.4. Predictive Models for Different Phenotypes of HF

**Figure 4:**
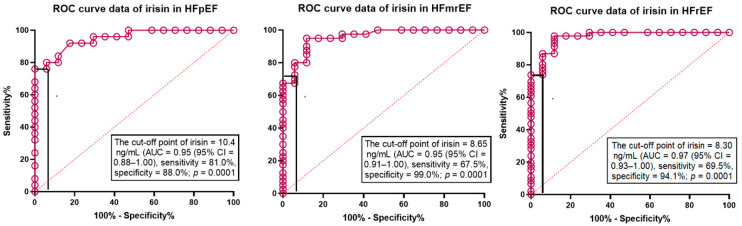
The predictive models based on the serum levels of irisin for different phenotypes of HF: The results of the ROC curve analysis. Abbreviations: AUC, area under curve; CI, confidence interval; HFpEF, heart failure with preserved ejection fraction; HFmrEF, heart failure with mildly reduced ejection fraction; HFrEF, heart failure with reduced ejection fraction.

The ROC curve analysis (Figure 4) showed that calculated cut-off points for serum concentration of irisin (against non-HF T2DM for all variables) were 10.4 ng/mL (area under curve [AUC] = 0.95 (95% CI = 0.88–1.00), sensitivity = 81.0%, specificity = 88.0%; Likelihood ratio = 6.881; *p* = 0.0001) in patients with HFpEF, 8.65 ng/mL (AUC = 0.95, sensitivity = 67.5%, specificity = 99.0%; Likelihood ratio = 11.48; *p* = 0.0001) in patients with HFmrEF and 8.30 ng/mL (AUC = 0.87, sensitivity = 69.5%, specificity = 94.1%; Likelihood ratio = 12.57; *p* = 0.0001) in HFrEF patients.

Univariate logistic regression showed that irisin < 10.4 ng/mL (OR = 1.52; *p* = 0.001), LV hypertrophy (OR = 1.12; *p* = 0.044), BMI > 34 kg/m^2^ (OR = 1.07; *p* = 0.046), NT-proBNP > 750 pmol/mL (OR = 1.54; *p* = 0.001), age (OR = 1.03; *p* = 0.048), LAVI > 34 mL/m^2^ (OR = 1.20; *p* = 0.001) and E/e’ > 11 (OR = 1.12; *p* = 0.001) were independent predictors for HFpEF in T2DM patients (Table 2). Pharmacological agents were predictors for the dependent variable. The multivariate logistic model yielded that the serum levels of irisin < 10.4 ng/mL (OR = 1.30; *p* = 0.001), NT-proBNP > 750 pmol/mL (OR = 1.17; *p* = 0.042) and LAVI > 34 mL/m^2^ (OR = 1.06; *p* = 0.042) remained strong predictors for HFpEF.

Levels of serum irisin < 8.65 ng/mL (OR = 1.37; *p* = 0.001), NT-proBNP > 2450 pmol/mL (OR = 1.46; *p* = 0.001), LV hypertrophy (OR = 1.09, *p* = 0.001) and LAVI > 34 mL/m^2^ (OR = 1.10; *p* = 0.001) were predictors for HFmrEF in the univariate logistic regression model, whereas multivariate logistic regression showed that irisin < 8.65 ng/mL (OR = 1.14; *p* = 0.045), NT-proBNP > 2450 pmol/mL (OR = 1.47; *p* = 0.001) and LAVI > 34 mL/m^2^ (OR = 1.10; *p* = 0.001) had independent discriminatory abilities for HFmrEF.

In addition, univariate logistic regression unveiled that HFrEF was predicted by the following variables, such as serum irisin < 8.65 ng/mL (OR = 1.38; *p* = 0.001), NT-proBNP > 2450 pmol/mL (OR = 1.54; *p* = 0.001), LAVI > 34 mL/m^2^ (OR = 1.11; *p* = 0.001) and eGFR (OR = 1.07; *p* = 0.042). Multivariate logistic regression showed that these variables, apart from LV hypertrophy and eGFR, remained predictors for HFrEF.

### 3.5. Comparison of the Predictive Models

Table 3 illustrates the fact that adding irisin < 10.4 ng/mL to the predictive model (NT-proBNP > 750 pg/mL) significantly improved the discriminatory potency of the whole model for HFpEF. When adding irisin < 8.65 ng/mL and irisin < 8.30 ng/mL to the based model (NT-proBNP > 2450 pg/mL), this constellation did not increase the predictive value of the whole model for both HfmrEF and HfrEF. Thus, irisin < 10.4 ng/mL noticeably improved the predictive ability of NTproBNP for HFpEF, but lowered levels of this biomarker failed to provide additional discriminatory information to NT-proBNP for HFmrEF/HFrEF.

## 4. Discussion

The results of the study reveal that different levels of irisin in T2DM patients seem to be predictive indicators for different phenotypes of HF. Indeed, there was a significant difference between the serum levels of irisin in HFpEF and HFrEF/HFmrEF in T2DM patients, whereas serum concentrations of NT-proBNP provided sufficient discrimination between the different phenotypes of HF. We also detected that, when adding irisin < 10.4 ng/mL to NT-proBNP, the total discriminatory potency of the whole model for HFpEF increased, whereas the predictive values of irisin and NT-proBNP for HFrEF and HFmrEF were fairly similar.

Although some circulating biomarkers (soluble suppressor tumorigenicity-2, galectin-3) may offer new opportunities to improve clinical outcomes in HF patients, in most cases, they do not have the predictive value of elevated NT-proBNP levels in patients with symptomatic HF of diverse phenotypes. A selective patient population, such as those with cachexia, T2DM, or chronic kidney disease [36,37,38], also presents challenges in this regard. On this occasion, irisin was a promising indicator of CV events [39,40].

However, serum levels of irisin is related to the spectrum of CV and metabolic diseases [14,17,21,22,23,24,26]. In fact, increased irisin levels have been found in the early stages of acute HF, acute myocardial infarction and unstable angina, whereas individuals with prediabetes/T2DM and chronic kidney disease demonstrated less dramatic changes in the irisin levels when compared with HF patients [12,17,41,42]. Numerous researchers believe that it provides adaptive protection of target organs, mainly heart, lung, kidney, brain and muscle, by reducing endothelial damage, inhibiting inflammation and suppressing oxidative stress and apoptosis [26,43,44,45].

We noticed that serum irisin levels were associated with the presence of all phenotypes of HF, and first established that only in HFpEF patients did irisin levels being added to the predictive model increase the discriminatory value of NT-proBNP. Previous studies determined that circulating levels of irisin demonstrated a divergent trend to be changed in patients with acute and chronic HF and that this was an obstacle to using it as a predictive biomarker. Indeed, in patients with acute myocardial infarction and acute HF, serum levels of irisin were significantly increased, whereas in chronic HF, they progressively decreased depending on the severity of the condition [24,25,26,46]. However, a few studies confirmed close positive linear correlations between serum levels of irisin and LVEF regardless of phenotypes of HF [23,47]. More often than not, irisin predicted reduced LVEF in chronic HF patients, whereas in acute HF, its predictive ability for short-term clinical outcomes was not related to LVEF [25,46,47].

It is challenging to identify primary mechanisms by which irisin maintains altered cardiac function and prevents adverse cardiac remodeling. This can be interpreted as depletion of numerous adaptive mechanisms regulating metabolic balance, skeletal muscle and adipose tissue energy consumption, endothelial progenitor cell differentiation and proliferation, and cardiac remodeling via PI3K/Akt/eNOS, AMPK-Akt-eNOS-NO, MAPK/p38, AMPK/mTOR, and uncoupling protein 1-related signaling mechanisms [46,47]. A potential explanation for these facts might relate to the catabolic state frequently observed in HFrEF and HFmrEF, and rarely in HFpEF [47]. Perhaps, deficiency of irisin directly causes a reduced autophagy and aggravated autophagy, leading to maladaptive cardiac remodeling and HF, whereas over-expressed irisin results in protective autophagy and improved autophagy flux [48,49]. Equally, it is conceivable that an altered metabolism results in a more efficient myocardial contraction and relaxation. The most important aggravating factor mediating irisin synthesis and release is T2DM and insulin resistance (IR) [50]. Additionally, T2DM plays a key role in stimulating microvascular inflammation, accelerating atherosclerosis, mediating mitochondrial dysfunction and adverse cardiac remodeling through oxidative stress, glucose and lipid toxicity, and dysregulation of the endogenous repair system promoting HF manifestation [51,52,53,54]. In this context, decreased irisin levels could serve as a predictive biomarker for an unfavorable stage in the regulation of cardiac function and the occurrence of HF.

In the present article, we describe for the first time that a decreased irisin level in T2DM patients was an independent predictor of all HF phenotypes. However, the cut-off values for distinguishing different HF phenotypes are different. In addition, we found that serum levels of irisin correlated positively with HOMA levels and negatively with BMI, parameters of glucose metabolism and BP in patients with HFpEF, but not in HFrEF or HFmrEF. The data correspond well with the result of a small study from Silvestrini et al., 2019 [55], supporting the hypothesis that different pathophysiological mechanisms are involved in the development of HF phenotypes in T2DM. Therefore, these findings may relate to the study population. Indeed, we did not include patients with a very high risk of hospitalization during HF progression or patients with NYHA IV, severe kidney dysfunction, recent acute MI, morbid obesity, as well as a permanent form of atrial fibrillation. In addition, we also did not enroll asymptomatic HF patients. The results of the study highlight the context of irisin serum levels with different phenotypes of HF in case of a stable euvolemic state, rather than the causes of HF with associated T2DM.

Unfortunately, there are limited data associated with various controversial findings regarding the short- and long-term monitoring of irisin and the therapeutic effect on its serum levels depending on the etiology of HF and coexisting comorbidities, including T2DM, obesity and stable coronary artery disease [56,57]. Cumulatively, serum levels of irisin grew dramatically in acute myocardial damage and acute HF, while biomechanical stress related to volume overload, chronic ischemia, hypertension and T2DM is associated with reduced concentrations of irisin in circulation.

To update our knowledge, the levels of irisin are regarded to be an attribute of the management of T2DM and HF. For instance, previous clinical studies have shown some benefit in cardiac myocyte protection due to an SGLT2 inhibitor-related increase in serum levels of various adipomyokines, such as apelin and irisin, and a decrease in myostatin in HF [58,59,60].

The results of the study show that extremely elevated circulating NT-proBNP concentrations had a predictive value in HFrEF and HFmrEF. However, the findings had a strict similarity to those previously reported [24,59,61]. Indeed, irisin demonstrated its predictive potency for HFpEF and indicated a possibility of improving the predictive ability of NT-proBNP during a period of slightly elevated concentrations, which could have serious clinical significance in T2DM with abdominal obesity, in whom natriuretic peptide levels appeared to be lower than in patients with normal or near-normal body weight. We suggest that the predictive value of increased NT-proBNP concentrations for HFrEF and HFmrEF in T2DM does not need to be re-evaluated, but adding irisin to the model seems intriguing and warrants further investigation in the future.

Overall, irisin deserves to be evaluated as a promising circulating biomarker with a potential additive predictive ability for HF, regardless of its phenotype, perhaps allowing the stratification of T2DM patients at risk without additional costs and the corresponding high expenditure of medical resources.

## 5. Study Limitations

The study has several limitations. The first limitation relates to the fact that this was a single-center study, in which T2DM patients were enrolled with the aim of pre-screening with further assessment in detail. Although the study protocol ensured a prospective inclusion of the patients, we had to minimize statistical bias through the non-inclusion of T2DM patients with a permanent form of atrial fibrillation or a history of TIA/stroke. Thus, a number of patients at high risk of an untoward clinical course were not enrolled in the study. The second limitation was that the detection of HF phenotypes was pre-determined by the measurement of LVEF and diastolic abnormalities, including LAVI, whereas NT-proBNP levels were mandatorily determined to exclude HFpEF. However, NT-proBNP levels were measured in all patients enrolled in the study, with initial selection. This is one reason why the predictive values of the biomarker were mandatorily elevated in patients with HFrEF and HFmrEF. Another limitation may be the relatively small size of each HF phenotype cohort. The final limitation was that we could not perform serial measurements of circulating biomarkers, as well as cardiac characteristics, to unambiguously assess the dynamics of these parameters, because the study design was based on a single examination. In the future, we will extend the assessment of patients at a 1-year follow-up to investigate whether the predictive value of irisin and NT-proBNP maintains their significance for different phenotypes of HF in T2DM patients.

## 6. Conclusions

We found that, in T2DM patients, serum irisin levels were associated with the presence of all phenotypes of HF, but only in HFpEF patients did irisin add a discriminatory value to NT-proBNP. This finding may open a new approach to HF risk stratification in T2DM patients.

## Figures and Tables

**Table 1:** Basic demographic, clinical, hemodynamic characteristics, biomarkers, biochemistry and concomitant medications of the study’s patient population.

Variables	Healthy Volunteers (*n* = 25)	Entire Patient Cohort (*n* = 183)	T2DM Patients (*n* = 183)				*p* Value
			HfpEF (*n* = 48)	HFmrEF (*n* = 49)	HFrEF (*n* = 56)	Non-HF (*n* = 30)	
Age, year	48 (42–55)	51 (41–62)	52 (43–62)	52 (41–64)	53(42–60)	51(41–60)	0.86
Male, *n* (%)	17 (68.0)	118 (64.5)	31 (64.6)	32 (65.3)	37 (66.1)	18 (60.0)	0.82
Dyslipidemia, *n* (%)	0	152 (83.1) #	38 (79.2)	41 (83.7)	48 (85.7)	25 (83.3)	0.82
Hypertension, *n* (%)	0	158 (86.3) #	43 (89.5)	39 (79.6)	50 (89.2)	26 (86.7)	0.79
Smoking, *n* (%)	5 (20.0)	89 (48.6) #	21 (43.8)	25 (51.0)	27 (48.2)	16 (53.3)	0.05
Abdominal obesity, *n* (%)	0	84 (45.9) #	22 (45.8)	24 (48.9)	25 (44.6)	13 (43.3)	0.88
Microalbuminuria, *n* (%)	0	56 (30.6) #	14 (29.1)	16 (32.7)	17 (30.4)	9 (30.0)	0.84
LV hypertrophy, *n* (%)	0	144 (78.7) #	41 (85.4)	39 (79.6)	43 (78.8)	21 (70.0)	0.001
BMI, kg/m^2^	21.9 ± 0.5	25.8 ± 2.1 #	25.5 ± 2.4	25.6 ± 2.8	25.2 ± 2.1	26.3 ± 2.6	0.88
Waist circumference, sm	75.0 ± 2.6	85.6 ± 2.9 #	85.4 ± 3.2	85.1 ± 3.2	85.0 ± 3.4	86.5 ± 3.1	0.86
WHR, units	0.78 ± 0.02	0.86 ± 0.03 #	0.85 ± 0.07	0.85 ± 0.05	0.84 ± 0.04	0.87 ± 0.03	0.86
II/III NYHA class, *n*	0	103/50 #	31/17	30/19	42/14	-	0.14
SBP, mm Hg	127 ± 4	132 ± 5	130 ± 4	130 ± 6	128 ± 5	135 ± 5	0.81
DBP, mm Hg	75 ± 3	80 ± 4	78 ± 4	76 ± 5	74 ± 4	84 ± 3	0.80
LVEDV, mL	88 ± 4	154 ± 9 #	159 ± 5	161 ± 4	162 ± 8	147 ± 6	0.001
LVESV, mL	30 ± 3	62 ± 7 #	66 ± 4	86 ± 6	104 ± 4	59 ± 3	0.001
LVEF, %	66 ± 2	59 ± 6 #	58 ± 3	46 ± 3	35 ± 4	60 ± 2	0.001
LVMMI, g/m^2^	80.7 ± 0.06	151 ± 6.12#	149 ± 4	154 ± 5	156 ± 7	137 ± 3	0.01
LAVI, mL/m^2^	22 ± 4	39 ± 8 #	36 ± 4	38 ± 4	41 ± 3	30 ± 5	0.03
E/e’, unit	5.4 ± 0.1	13.9 ± 0.5 #	12.8 ± 0.2	13.5 ± 0.3	15.1 ± 0.3	7.2 ± 0.4	0.001
eGFR, mL/min/1.73 m^2^	108 ± 5.10	83 ± 6.0 #	81 ± 4.2	75 ± 4.0	73 ± 3.5	86 ± 3.5	0.01
HOMA-IR	1.53 ± 0.30	7.65 ± 3.7 #	7.90 ± 3.0	7.95 ± 2.3	8.02 ± 2.1	7.15 ± 2.4	0.14
NT-proBNP, pmol/mL	52 (33–74)	2718 (1380–3720) #	998 (745–1126)	3115 (2380–3750)	3125 (2540–3810)	105 (72–142)	0.001
Fasting glucose, mmol/L	4.22 ± 0.70	5.84 ± 1.2 #	5.70 ± 1.5	5.62 ± 1.3	5.45 ± 1.2	5.92 ± 1.3	0.28
Creatinine, mcmol/L	52.5 ± 9.3	108.8 ± 12.0 #	103.7 ± 9.8	108.6 ± 8.5	112.5 ± 6.1	95.1 ± 10.4	0.26
HbA1c, %	4.20 ± 0.95	6.65 ± 0.04 #	6.54 ± 0.03	6.59 ± 0.02	6.55 ± 0.03	6.70 ± 0.05	0.70
TC, mmol/L	4.6 ± 0.09	6.41 ± 0.05 #	6.37 ± 0.68	6.43 ± 0.60	6.40 ± 0.46	6.42 ± 0.55	0.82
HDL-C, mmol/L	1.2 ± 0.03	0.95 ± 0.21 #	0.97 ± 0.22	0.97 ± 0.17	0.95 ± 0.14	0.93 ± 0.24	0.80
LDL-C, mmol/L	2.8 ± 0.05	4.43 ± 0.20 #	4.42 ± 0.12	4.38 ± 0.10	4.35 ± 0.11	4.51 ± 0.15	0.68
TG, mmol/L	1.3 ± 0.04	2.26 ± 0.04 #	2.23 ± 0.19	2.21 ± 0.17	2.20 ± 0.12	2.30 ± 1.12	0.64
SGLT2i, *n* (%)	0	171 (93.4)	48 (100)	49 (100)	56 (100)	18 (60)	0.82
ACEIs/ARBs/ARNI, *n* (%)	0	158 (86.3) #	43 (89.5)	39 (79.6)	50 (89.2)	26 (86.7)	0.80

Notes: data of variables are given as the mean ± SD and median (interquartile range), #—significant difference between healthy volunteers and entire T2DM cohort. Variables were compared with the Tukey test. Abbreviations: BMI, body mass index; DBP, diastolic blood pressure; E/e’, early diastolic blood filling to longitudinal strain ratio; GFR, glomerular filtration rate; LDL-C, low-density lipoprotein cholesterol; HbA1c, glycosylated hemoglobin; HDL-C, high-density lipoprotein cholesterol; HF, heart failure; HOMA-IR, Homeostatic Assessment Model of Insulin Resistance; HFpEF, heart failure with preserved ejection fraction; HFmrEF, heart failure with mildly reduced ejection fraction; HFrEF, heart failure with reduced ejection fraction; NT-proBNP, N-terminal brain natriuretic pro-peptide; LVEDV, left ventricular end-diastolic volume; LVESV, left ventricular end-systolic volume; LVEF, left ventricular ejection fraction; LVMMI, left ventricle myocardial mass index; LAVI, left atrial volume index; SBP, systolic blood pressure; SGLT2i, sodium-glucose cotransporter-2 inhibitor; T2DM, type 2 diabetes mellitus; TG, triglycerides; TC, total cholesterol; WHR, waist-to-hip ratio.

**Table 2:** Predictors for dependent variables (HFpEF, HFmrEF and HFrEF) in T2DM populations. The results of the univariate and multivariate log regression analysis.

Variables	Dependent Variables					
	Univariate Log Regression			Multivariate Log Regression		
	OR	95% CI	*p*-Value	OR	95% CI	*p*-Value
Dependent variable: HFpEF						
Irisin < 10.4 ng/mL	1.52	1.16–2.86	0.001	1.30	1.08–2.15	0.001
LV hypertrophy	1.12	1.06–1.19	0.044	1.05	1.00–1.11	0.14
eGFR	0.93	0.89–1.02	0.94	-		
BMI > 34 kg/m^2^	1.07	1.02–1.11	0.046	1.05	1.00–1.08	0.062
NT-proBNP > 750 pmol/mL	1.54	1.06–2.33	0.001	1.17	1.02–1.26	0.042
Age	1.03	1.02–1.05	0.048	1.03	1.00–1.04	0.16
Smoking	1.04	0.98–1.07	0.92	-		
E/e’ > 11 units	1.12	1.06–1.20	0.001	1.04	1.00–1.06	0.42
LAVI > 34 mL/m^2^	1.20	1.11–1.36	0.001	1.06	1.02–1.13	0.042
Dependent variable: HFmrEF						
Irisin < 8.65 ng/mL	1.37	1.12–1.55	0.001	1.14	1.02–1.77	0.045
NT-proBNP > 2450 pmol/mL	1.46	1.16–2.33	0.001	1.47	1.22–2.66	0.001
LV hypertrophy	1.09	1.02–1.15	0.001	1.07	1.00–1.12	0.62
E/e’ > 11 units	1.02	1.00–1.05	0.92	-		
LAVI > 34 mL/m^2^	1.10	1.02–1.17	0.001	1.08	1.02–1.19	0.014
Dependent variable: HFrEF						
Irisin < 8.30 ng/mL	1.38	1.17–1.62	0.001	1.19	1.05–1.30	0.001
NT-proBNP > 2450 pmol/mL	1.54	1.14–2.70	0.001	1.47	1.22–2.66	0.001
LV hypertrophy	1.06	1.00–1.12	0.86	-		
LAVI > 34 mL/m^2^	1.11	1.01–1.15	0.048	1.09	1.02–1.16	0.010
eGFR	1.07	1.02–1.14	0.042	1.05	1.00–1.09	0.058

Abbreviations: ARBs, angiotensin-II receptor blockers; ARNI, angiotensin receptor neprilysin inhibitor; ACEIs, angiotensin-converting enzyme inhibitors; BMI, body mass index; CI, confidence interval; eGFR, estimated glomerular filtration rate; NT-proBNP, N-terminal brain natriuretic pro-peptide; LV, left ventricle; LAVI; left atrial volume index; E/e’, early diastolic blood filling to longitudinal strain ratio; HFpEF, heart failure with preserved ejection fraction; HFmrEF, heart failure with mildly reduced ejection fraction; HFrEF, heart failure with reduced ejection fraction; SGLT2i, sodium-glucose co-transporter 2-inhibitors; OR, odds ratio.

**Table 3:** The comparisons of predictive models for HF: The results of statistics for model fit.

Predictive Models	Dependent Variable: HF					
	AUC		NRI		IDI	
	M (95% CI)	*p* Value	M (95% CI)	*p* Value	M (95% CI)	*p* Value
Dependent variable: HFpEF						
Model 1 (NT-proBNP > 750 pg/mL)	0.70 (0.63–0.76)	-	Reference	-	Reference	-
Model 2 (NT-proBNP > 750 pg/mL + irisin < 10.4 ng/mL)	0.85 (0.78–0.92)	0.001	0.63 (0.61–0.66)	0.045	0.56 (0.51–0.60)	0.012
Dependent variable: HFmrEF						
Model 1 (NT-proBNP > 2450 pg/mL)	0.76 (0.68–0.85)	-	Reference	-	Reference	-
Model 2 (NT-proBNP > 750 pg/mL + irisin < 8.65 ng/mL)	0.79 (0.65–0.90)	0.16	0.35 (0.33–0.38)	0.28	0.22 (0.21–0.24)	0.66
Dependent variable: HFmrEF						
Model 1 (NT-proBNP > 2450 pg/mL)	0.85 (0.76–0.94)	-	Reference	-	Reference	-
Model 2 (NT-proBNP > 750 pg/mL + irisin < 8.30 ng/mL)	0.87 (0.79–0.95)	0.64	0.35 (0.33–0.38)	0.66	0.27 (0.22–0.31)	0.72

Abbreviations: AUC, area under curve; NT-proBNP, N-terminal brain natriuretic pro-peptide; HF, heart failure; HFpEF, heart failure with preserved ejection fraction; HFmrEF, heart failure with mildly reduced ejection fraction; HFrEF, heart failure with reduced ejection fraction; IDI, integrated discrimination indices; NRI, net reclassification improvement.

## Data Availability

Not applicable.
